# Multi‐omics data identified TP53 and LRP1B as key regulatory gene related to immune phenotypes via EPCAM in HCC

**DOI:** 10.1002/cam4.4594

**Published:** 2022-02-12

**Authors:** Liang Shi, Jie Cao, Xin Lei, Yifen Shi, Lili Wu

**Affiliations:** ^1^ Department of Clinical Laboratory Medicine, The Eighth Affiliated Hospital Sun Yat‐sen University Shenzhen China; ^2^ Translational Medicine Laboratory The First Affiliated Hospital of Wenzhou Medical University Wenzhou China; ^3^ Department of Hematology The First Affiliated Hospital of Wenzhou Medical University Wenzhou China; ^4^ Department of Clinical Blood Transfusion, The Eighth Affiliated Hospital Sun Yat‐sen University Shenzhen China; ^5^ Department of Clinical Laboratory The Central Hospital of Wenzhou Wenzhou China

**Keywords:** hepatocellular carcinoma, immune phenotypes, LRP1B, prognosis, TP53

## Abstract

**Background:**

Many studies showed that the prognosis of hepatocellular carcinoma (HCC) was significantly associated with the expressions of TP53 and LRP1B. However, the potential influence of the two genes on the malignant progression of HCC is still to be expounded.

**Methods:**

According to the correlation analysis between immune cells and expression levels of TP53 and LRP1B, we filtered the immune cells to perform unsupervised clustering analysis. Integration of multi‐omic data analysis identified genetic alteration and epigenetic alteration. In addition, pathway analysis was used to explore the potential function of the differentially expressed mRNAs. According to the differentially expressed genes, we established an interaction network to seek the hub gene. Least absolute shrinkage and selection operator (LASSO) regression analysis was used to build a prognosis model.

**Results:**

The unsupervised clustering analysis showed that the cluster A1 showed the highest immune cell levels and the cluster B2 showed the lowest immune cell levels. Multi‐omics data analysis identified that somatic mutations, copy number variations, and DNA methylation levels had significant differences between cluster A1 and cluster B2. Gene Ontology (GO) and Kyoto Encyclopedia of Genes and Genomes (KEGG) analysis found that the upregulated mRNAs in the cluster A1 were mainly concentrated in T cell activation, external side of plasma membrane, receptor ligand activity, and cytokine−cytokine receptor interaction. Importantly, the EPCAM was identified as a critical node in the lncRNAs–miRNAs–mRNAs regulatory network correlated with the immune phenotypes. In addition, based on differentially expressed genes between cluster A1 and cluster B2, the prognostic model established by LASSO could predict the overall survival (OS) of HCC accurately.

**Conclusions:**

The results indicated that the TP53 and LRP1B acted as the key genes in regulating the immune phenotypes of HCC via EPCAM.

## Graphical abstract

A new molecular classifier associated with the expression of TP53 and LRP1B was developed, which displayed different subgroups with higher or lower immune cell levels. Integration of multi‐omic data analysis identified genetic alteration and epigenetic alteration between the two subgroups. The prognostic model was established to predict the overall survival of HCC accurately.

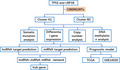

## 1 INTRODUCTION

Hepatocellular carcinoma (HCC), a main reason for cancer death in the world, is the most important pathological classification of liver cancer, comprising 75%–85% of the proportion.^1^ With the rapid development of the sequencing technologies,^2^ many studies have revealed that some genes significantly affected the prognosis of HCC patients.^3^ However, the molecular pathological mechanisms underlying the development of HCC are still unclear.

Mutation of TP53, as the antioncogene, is the most frequent gene change of HCC patients, and the TP53 mutation frequency is about 30%.^4^ Based on the DNA destruction events, the cells with TP53 mutation can be able to evade apoptosis and turn into the cancer cells.^5^ Some studies have shown that the TP53 gene was relevant to the levels of serum alpha‐fetoprotein (AFP), vascular invasion, tumor stage, and tumor differentiation of HCC patients, while at the same time it influenced the prognosis.^6^ In addition, TP53 mutation is extremely correlative with the tumor immune microenvironment of HCC and the different mutation status of TP53 is associated with immune responses.^7^

The LRP1B which is the LDL receptor family member is the significantly important tumor suppressor gene. The expression level of LRP1B is reduced in many tumors and it is among the top 10 mutated genes in the tumor.^8^ The inactivation of the LRP1B promotes cell migration and invasion and the genomic deletion of LRP1B exerts the poor prognosis of patients.^9^, ^10^ The inactivation of LRP1B may also bring about the alteration of the tumor microenvironment that could confer the increased tumor growth and enhanced tumor invasion ability.^11^ In recent years, LRP1B has been proved to participate the antigen presentation and served as a regulatory factor of tumor progression and inflammation.^12^, ^13^

In addition, many studies revealed that the presence of synchronous mutations of TP53 and LRP1B was found in different cancers.^14^ Research has shown that TP53 and LRP1B mutations acted as the prognostic biomarkers and related with higher TMB, which could predict the efficacy of immunotherapy in HCC patients.^15^ Besides, LRP1B mutation and TP53 mutation were significantly correlated with proportions of tumor‐infiltrating immune cells in esophageal cancer (EC).^16^ The above studies indicated that TP53 and LRP1B could affect the tumor immune microenvironment (TIME), but the potential mechanism has not been expounded in the detail.

The TIME, acting as a dynamic and complex system, is consisted of immune cells, immune matrix, and stromal cells.^17^ In the tumor microenvironment, immune cell imbalance is the crucial factor of HCC progression. Some studies have demonstrated that the TIME could regulate populations of immune and host cells to affect tumor prognosis.^18^ Importantly, the TIME immune inhibition on tumors is controlled with immune cells, including T lymphocytes, B lymphocytes, natural killer cells, dendritic cells, and macrophages. Nevertheless, immune cells, such as macrophages and regulatory T cells, accelerate tumor progression. These immune cell components provide biomarkers of diagnostic, prognostic, and immune therapy strategies for numerous patients with tumor, such as HCC patients.^19^

Because of the rapid development of the high‐throughput sequencing, abundantly biological data of diseases are available in the diverse databases at current. These biological data contain multiple omics data including proteomics, metabolomics, and transcriptomics data. Each of them represents the different field of cellular mechanisms.^20^ Based on the huge resources, we can discover the potential mechanisms of the various diseases and identify the dependable biomarkers to predict the prognosis. Multi‐omics data analysis can identify the relationship among multiple type biology factors. Compared with the single‐omics data analysis, multi‐omics data analysis has significant advantages in revealing the functional mechanisms and causes of complex diseases, providing a more well‐rounded description for biological processes and accelerating precision medicine progress.

In this study, we explored the association between immune cells and the expressions of TP53 and LRP1B. According to the immune cells, unsupervised clustering analysis divided patients into different groups. Furthermore, multi‐omics data analysis revealed the differences between two groups. A critical node of the interaction network was selected. In addition, based on the different immune status, we established the prognostic model for patients with HCC.

## 2 METHODS

### 2.1 Data source

**FIGURE 1:**
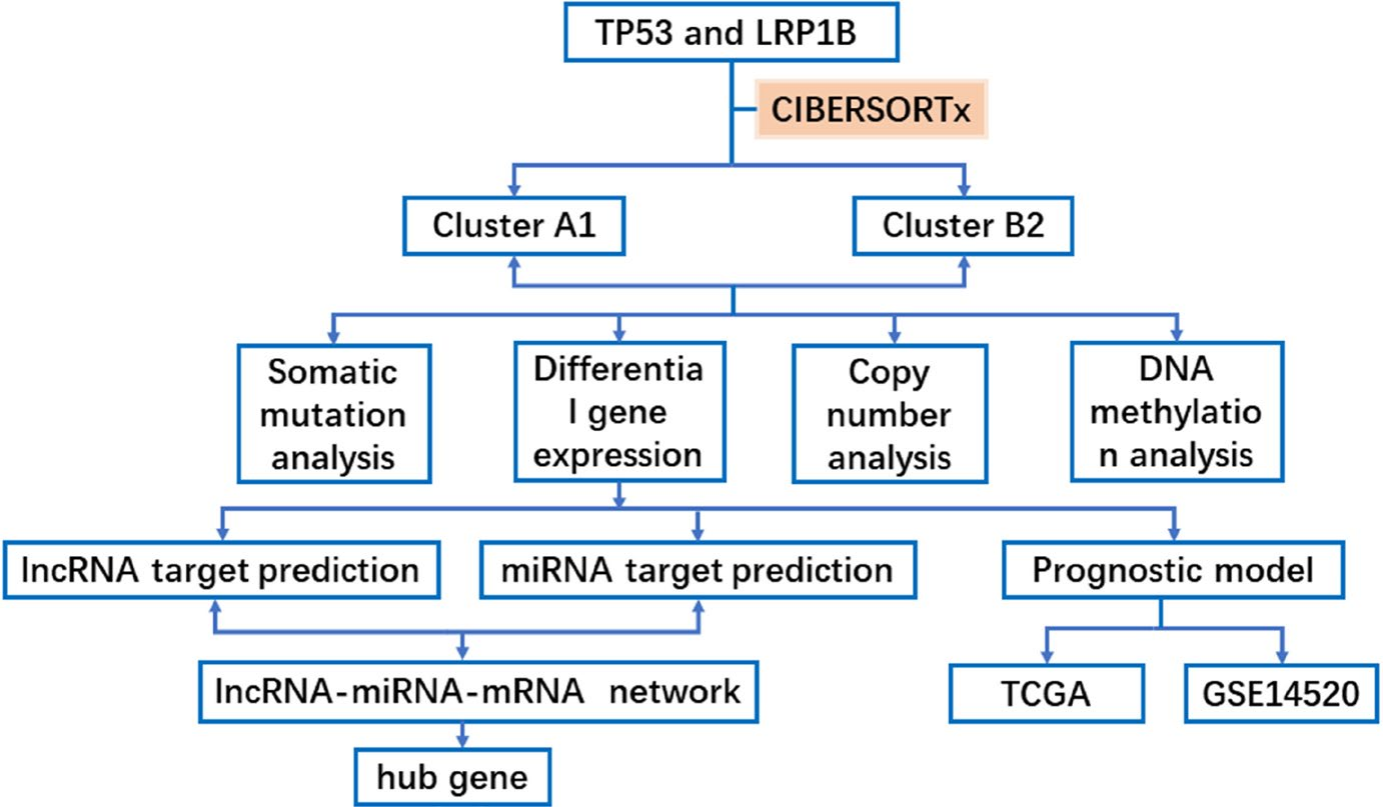
The flow chart showing the multi‐omic data analysis between cluster A1 and cluster B2, identification of hub gene and the establishment of prognosis model

The research design are shown in Figure 1. The clinical data and expression data of mRNAs, miRNAs, and lncRNAs were downloaded from The Cancer Genome Atlas (TCGA) database (https://portal.gdc.cancer.gov/), which acted as the training set and contained 374 HCC samples.^21^ The protein expression profiles for HCC patients were obtained from The Cancer Proteome Atlas (TCPA) database (https://www.tcpaportal.org/).^22^ In addition, three datasets including GSE14520, ICGC‐FR, and ICGC‐JP served as independent validation cohorts. GSE14520 included 247 HCC samples was downloaded from the NCBI Gene Expression Omnibus (GEO) database (https://www.ncbi.nlm.nih.gov/geo/).^23^ ICGC‐FR and ICGC‐JP datasets were obtained from The International Cancer Genome Consortium (ICGC) database (https://icgc.org/), thereinto, ICGC‐FR and ICGC‐JP datasets had 161 and 229 samples, separately (Tables S1 and S2).^24^

### 2.2 CIBERSORTx and clustering analyses

The CIBERSORT was used to estimate the cell‐type abundance from RNA‐seq data. According to the CIBERSORT, CIBERSORTx (https://cibersortx.stanford.edu/) was developed, which added the inference of expression level cell‐type‐specific gene. The CIBERSORTx “digitally purifies” individual cell types transcriptome from a large amount of data without requiring the isolation of single cells.^25^, ^26^ The expression profiles of TCGA, GEO, and ICGC were uploaded on the CIBERSORTx. The absolute immune cell scores were calculated by the LM22 (22 types of immune cells) gene signature of CIBERSORTx.^27^ The correlation analysis and least absolute shrinkage and selection operator (LASSO) regression analysis were used to filter the immune cells, which had high correlation with the gene expressions of TP53 and LRP1B.^28^ Based on the immune cells obtained above, the unsupervised cluster method was used to distinguish patients in the TCGA‐LIHC set into cluster A and cluster B. Then, the cluster A was subdivided into A1 and A2 clusters and cluster B was subdivided into B1, B2, and B3 clusters. Furthermore, we compared the levels of infiltrating immune cells in five clusters. The cluster A1 showed the highest immune cell levels and the cluster B2 showed the lowest immune cell levels. So, the cluster A1 and cluster B2 were selected to study the differences of HCC with high immunogenicity and low immunogenicity.

### 2.3 Somatic mutations analysis

The data of somatic mutations were downloaded from the UCSC Xena browser (https://xenabrowser.net/).^29^ The VarScan2 was used to analyze the somatic mutations of the HCC patients.^30^ Data were extracted by the R Studio (version:3.6.3). Subsequently, gene mutations, synergy, and mutual exclusion analysis were explored by maftools.^31^

### 2.4 Differential gene analysis and functional enrichment analyses

The differentially expressed mRNAs (padj<0.05, logFC>1 or logFC<−1), miRNAs (padj<0.05), and lncRNAs (padj<0.05, logFC>1 or logFC<−1) between cluster A1 and cluster B2 were screened by the Deseq2 package in R.^32^ The volcano plots of the differentially expressed genes were created. In order to explore the potential function of the differentially expressed genes, the functional enrichment analyses were proceeded by the clusterProfiler package.^33^ *p*_adjust_ < 0.01 was considered statistically significant.

### 2.5 Copy number variations analysis and DNA methylation analysis

Copy number variations and DNA methylation data were collected from the UCSC Xena. The genes with the different copy number variations were filtered by the *p* < 0.05. According to the IGV, we visualized the copy number variations and gene location information.^34^ For DNA methylation analysis, we employed k‐nearest neighboring (KNN) algorithm from the “impute” package to fill the missing values. Subsequently, the different methylation probes were detected by the “minfi” package in R.^35^

### 2.6 Construction of the lncRNAs–miRNAs–mRNAs network

The miRcode (http://www.mircode.org/) database was applied to predict the target miRNAs of the differentially expressed lncRNAs.^36^ The target mRNAs of miRNAs were predicted using TargetScan (http://www.targetscan.org/), miRDB (http://mirdb.org/), and miRTarbase (http://mirtarbase.cuhk.edu.cn/) databases.^37^, ^38^, ^39^ The predictive target mRNAs were displayed with the Venn diagrams. All target genes predicted using miRcode, TargatScan, miRDB, and miRTarbase databases were selected. The target networks simultaneously predicted by at least two databases acted as the significant regulation networks. Based on the lncRNAs–miRNAs and miRNAs–mRNAs networks and string database (https://string‐db.org/), the lncRNAs–miRNAs–mRNAs interaction network was constructed and visualized by the cytoscape.^40^, ^41^ In order to seek the core gene of the interaction network, we used the cytohubba plug‐in and EcCentricity topological feature.

### 2.7 Construction of the prognostic model

In order to filter the prognostic genes from the differentially expressed genes, univariate analysis was first used to select genes, which had significant correlations with overall survival (OS) (*p* < 0.005) (Table S3). Based on the “glmnet” package, the LASSO analysis was applied to construct a prognostic model.^42^ In addition, we used the GSE14520 to verify the availability of the prognostic model.

## 3 RESULTS

### 3.1 Immunophenotypes associated with TP53 and LRP1B expression in HCC

**FIGURE 2:**
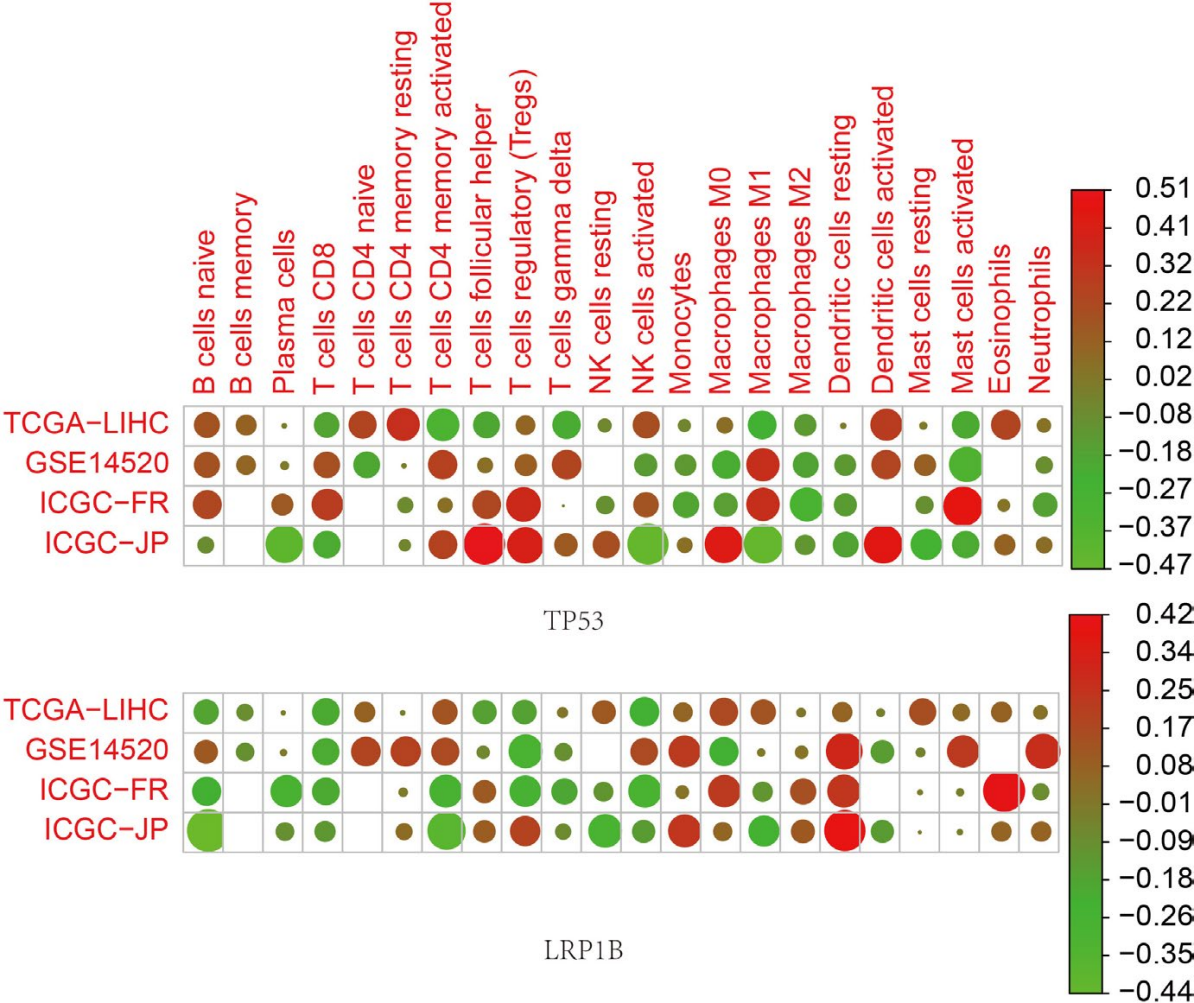
Correlation analysis between immune infiltrating cells and gene expressions and the size of the data points indicated significance

Four independent datasets of gene expression with 1011 samples were analyzed with CIBERSORTx to evaluate the proportions of 22 immune‐related cell subtypes of each patient. The correlations between the TP53 and LRP1B expressions levels and the proportions of immune cells were calculated by Spearman's analysis. Twelve immune cells (T cells CD4 memory resting, T cells CD4 memory activated, Dendritic cells activated, Macrophages M1, T cells CD4 naïve, Eosinophils, T cells gamma delta, Mast cells activated, NK cells activated, T cells follicular helper, B cells naïve, T cells CD8) had strong correlations with TP53 expression level. Eight immune cells (NK cells activated, Macrophages M0, T cells CD8, Mast cells resting, T cells CD4 memory activated, B cells naïve, Macrophages M1, T cells regulatory [Tregs]) had strong correlations with LRP1B expression level (Figure 2). LASSO regression analysis was used to further select the immune cells (T cells CD8, NK cells activated, T cells CD4 memory activated and Macrophages M1), which were associated with TP53 and LRP1B expression levels. In addition, we found that Macrophages M2 had a high correlation with TP53 expression level and M1/M2 immune cells represented two extreme directions of immune differentiation. So, Macrophages M2 was also selected. Ultimately, five immune cells including T cells CD8, NK cells activated, T cells CD4 memory activated, Macrophages M1 and Macrophages M2 were applied in the subsequent analysis.

### 3.2 Stratification of patients by five immune cells

**FIGURE 3:**
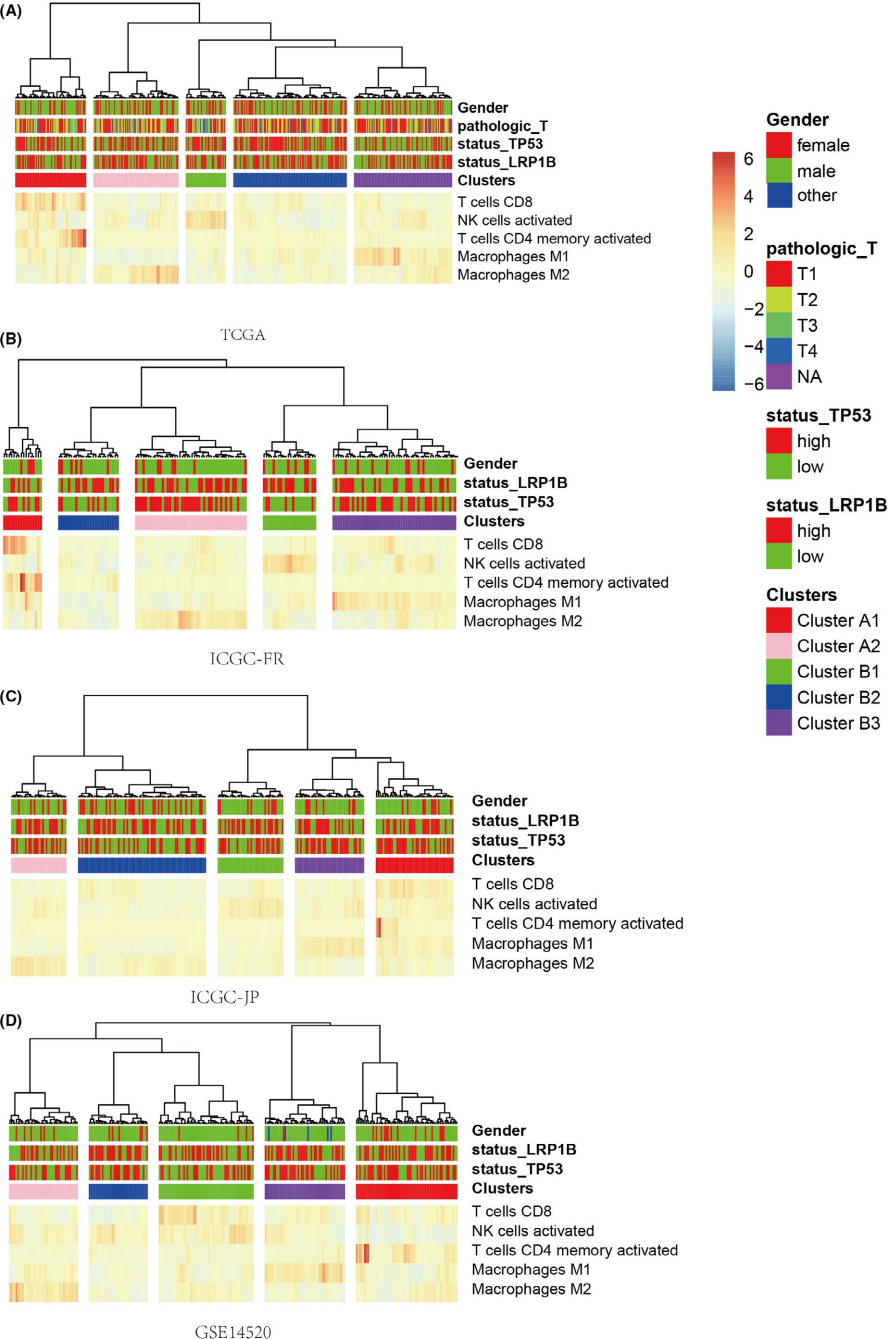
Based on immune cell proportion data, unsupervised cluster analysis of TCGA (A), ICGC‐FR (B), ICGC‐JP (C), and GSE14520 (D). TCGA, The Cancer Genome Atlas; ICGC‐FR, The International Cancer Genome Consortium‐France; ICGC‐JP, The International Cancer Genome Consortium‐Japan

According to the five immune cells selected above, the unsupervised clustering analysis of the TCGA‐LIHC set displayed two distinguished clusters including A and B (Figure 3A). The cluster A was separated into A1 and A2 subclusters and cluster B was separated into B1, B2, and B3 subclusters. The cluster A showed a higher proportion of infiltrating immune cells than the cluster B. In addition, the levels of T cells CD8 and T cells CD4 memory activate in cluster A1 were higher than the cluster A2, and the levels of Macrophages M2 in cluster A1 were lower than the cluster A2. The cluster B1 had a higher proportion of NK cells activated. The cluster B3 had a higher proportion of Macrophages M1, whereas various immune cells of cluster B2 showed lower levels. Furthermore, the similar stratification pattern of patients was verified in three independent validation sets (Figure 3B–D). Because of the highest immune cell levels of cluster A1 and lowest immune cell levels of cluster B2, we selected the cluster A1 and cluster B2 to study the differences of HCC with high immunogenicity and low immunogenicity. In order to study the difference of the TP53 in diverse clusters, we analyzed protein and transcript levels using the TCPA database and TCGA dataset. However, there was no significant difference between clusterA1 and cluster B2 (Figure S1A,B).

### 3.3 Differences of somatic mutations in the diverse immunophenotypes

**FIGURE 4:**
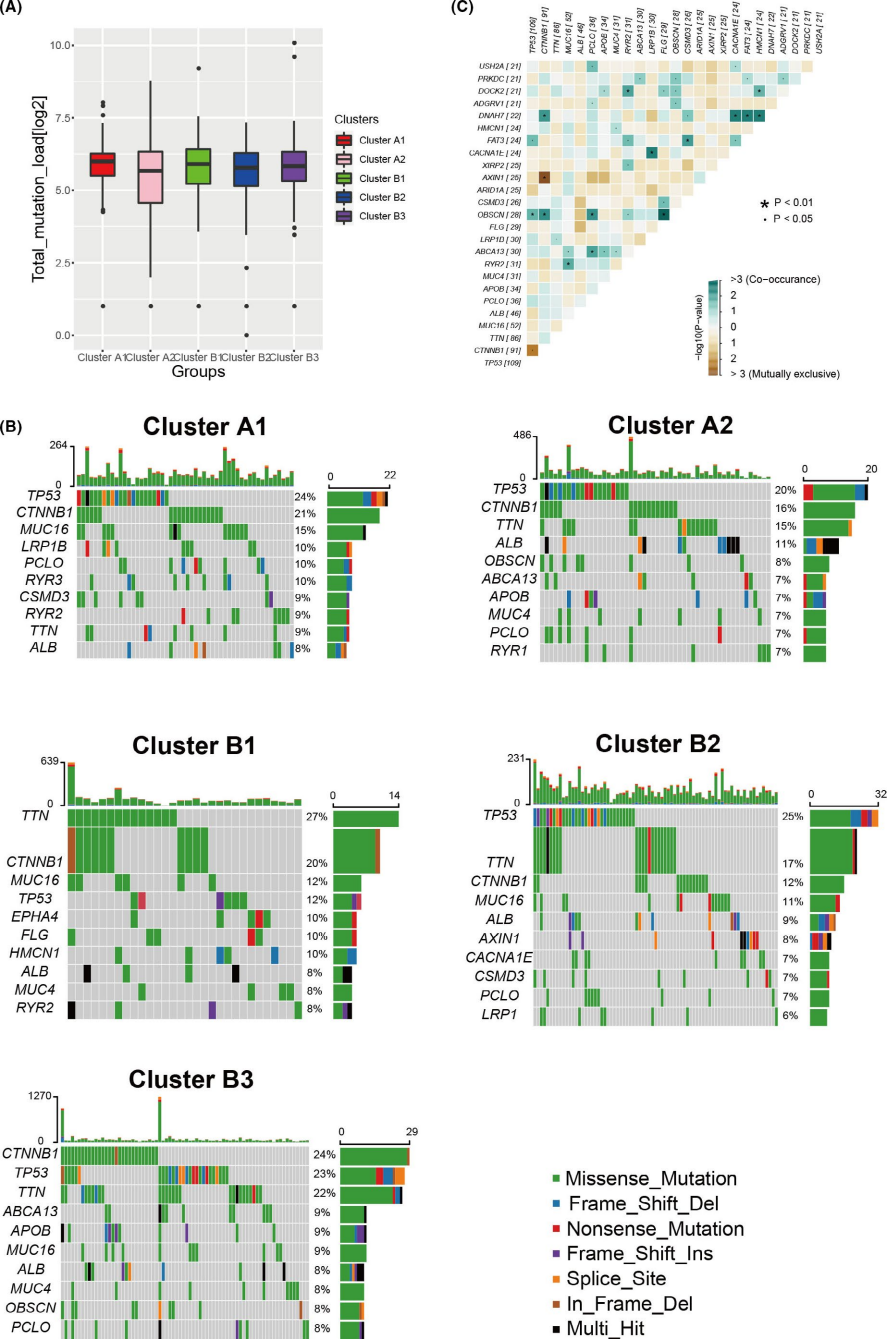
Somatic mutations analysis. (A) The total mutation rate of different groups. (B) Analysis of mutation types and mutation frequencies of different groups. The top 10 genes of mutation frequencies were visualized. (C) Synergy genes and mutual exclusion genes analysis of somatic mutations

To study the differences of somatic mutations in the diverse clusters, the proportion and quantity of somatic mutations of the TCGA‐LIHC set were analyzed. The synergy genes and mutual exclusion genes were researched at the same time. First, we analyzed the total mutation counts and found that there was no significant difference among different clusters, which indicated that the total mutation counts had no obvious relationships with immunophenotypes (Figure 4A). Moreover, the mutation frequency of TTN in cluster A1 was lower than other clusters and the LRP1B mutation was only found in cluster A1. The lower mutation frequency of TTN and higher mutation frequency of LRP1B might be associated with the high immunity of cluster A1. However, the high mutation frequencies of TP53 and CTNNB1 in all clusters showed that both genes existed commonly in HCC (Figure 4B). In addition, the mutation analysis found that the TP53 had the synergistic phenomenon with FAT3 and OBSCN, but had a mutually exclusive phenomenon with CTNNB1 (Figure 4C). Furthermore, LRP1B had the synergistic phenomenon with both genes of TTN and CACAN1E.

### 3.4 Differential expression of mRNAs and functional enrichment analyses

**FIGURE 5:**
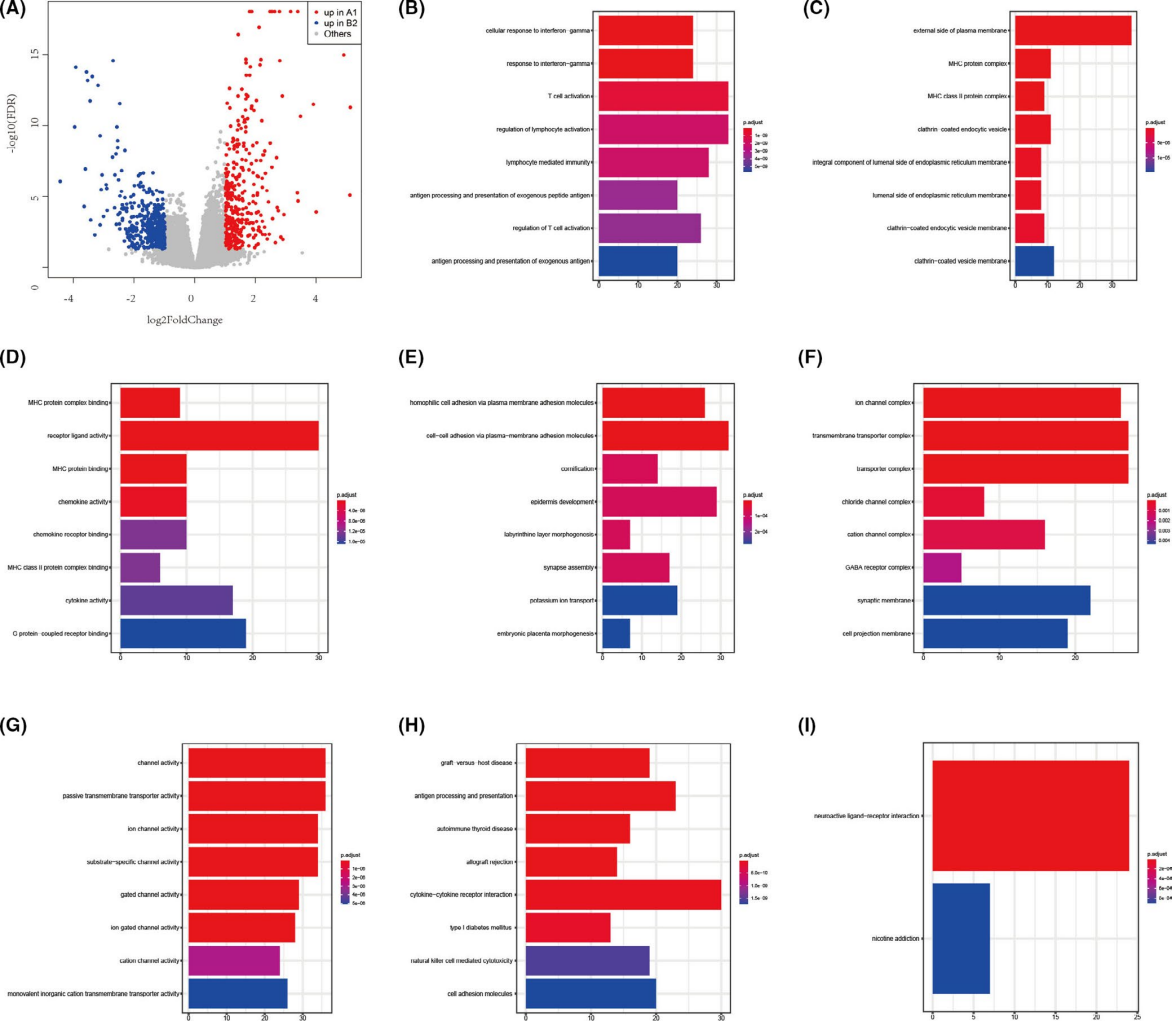
The differential expression of mRNAs and functional enrichment analyses. (A) The volcano plot of the differentially expressed mRNAs between cluster A1 and cluster B2. (B–G) The GO analysis: BP (B), CC (C), and MF (D) analysis of the upregulated differently expressed mRNAs in cluster A1; BP (E), CC (F), and MF (G) analysis of the downregulated differently expressed mRNAs in cluster A1. (H–I) The KEGG analysis: (H) KEGG analysis of the upregulated differently expressed mRNAs in cluster A1; (I) KEGG analysis of the downregulated differently expressed mRNAs in cluster A1. BP, biological process; CC, cellular component; GO, gene ontology; MF, molecular function; KEGG, Kyoto encyclopedia of genes and genomes

In order to further explore the differences between the cluster A1 and cluster B2, the differentially expressed genes were obtained from the TCGA‐LIHC set. In total, 798 differentially expressed genes were identified between two clusters (Figure 5A). Compared with cluster B2, 349 genes were significantly upregulated and 449 genes were significantly downregulated in the clusterA1. The Gene Ontology (GO) analysis and Kyoto Encyclopedia of Genes and Genomes (KEGG) analysis were used to analyze the potential function of the differentially expressed genes. The GO analysis included biological process (BP), cellular component (CC) and molecular function (MF) revealed that upregulated differentially expressed genes in cluster A1 were associated with T cell activation, external side of plasma membrane and receptor ligand activity, while downregulated differentially expressed genes in cluster A1 were associated with cell–cell adhesion via plasma membrane adhesion molecules, transmembrane transporter complex and channel activity (Figure 5B–G). The KEGG analysis revealed that upregulated differentially expressed genes in cluster A1 were associated with cytokine−cytokine receptor interaction, antigen processing and presentation and cell adhesion molecules (Figure 5H). We displayed eight pathways with the most significant *p*_adjust_. However, the KEGG analysis revealed that the downregulated differentially expressed genes in cluster A1 were enriched in two pathways, including neuroactive ligand−receptor interaction and nicotine addiction (Figure 5I).

### 3.5 Differences of copy number variations in the diverse immunophenotypes

Recently, Davoli et al. offered forceful evidence that the copy number variations were related to immune escape,^43^ which indicated that the genomic alterations could strongly impact the immunophenotypes of tumors. The genomic analysis showed that several regions (chromosomes 1, 16, and 21) had the significant differences of copy number variations between cluster A1 and cluster B2. In addition, we found 202 genes with copy number gains in cluster A1 and 814 genes with copy number gains in cluster B2. Thereinto, 5 of 202 genes were upregulated differentially expressed genes in cluster A1 and 26 of 814 genes were upregulated differentially expressed genes in cluster B2 (*p* < 0.05) (Figure S2A). Therefore, the copy number variations of these genes might be associated with the immunity of cluster A1 and cluster B2.

### 3.6 Differences of DNA methylation in the diverse immunophenotypes

To research the differences of the DNA methylation in the diverse immunophenotypes, we investigated the differentially methylated sites between cluster A1 and cluster B2. We found that 7068 methylation probes had significantly higher beta values in cluster A1 and 4045 methylation probes had significantly higher beta values in cluster B2 (*p* < 0.05). According to the theory that DNA methylation negatively regulated the expression of mRNAs, we found six differentially expressed mRNAs which showed high expression and low DNA methylation in cluster A1 while 71 differentially expressed mRNAs which showed high expression and low methylation in cluster B2 (Figure S2B).

### 3.7 Differential expression of miRNAs and lncRNAs

According to the study of the miRNAs, we identified 113 differentially expressed miRNAs between cluster A1 and cluster B2 of the TCGA‐LIHC set. Thereinto, 87 genes were significantly upregulated in cluster A1 and 26 genes were significantly upregulated in the cluster B2 (Figure S3A). We predicted the target genes of miRNA using three databases including TargetScan, miRDB, and miRTarbase databases. The target genes predicted simultaneously at least two databases were selected. Ultimately, 9532 target mRNAs were obtained for the upregulated differentially expressed miRNAs in the cluster A1 (Figure S3B), 1267 target mRNAs were obtained for the upregulated differentially expressed miRNAs in the cluster B2 (Figure S3C). Next, we identified 322 lncRNAs, which were differentially expressed between cluster A1 and cluster B2. One hundred and sixty‐five genes were upregulated in cluster A1, while 157 genes were upregulated in cluster B2 (Figure S3D). We predicted the target miRNAs of the differential expressed lncRNAs by miRcode database. According to TargetScan, miRDB, and miRTarbase database, we predicted the target mRNAs of the miRNAs. The target mRNAs simultaneously predicted by at least two databases were also filtered. Ultimately, 66 target mRNAs were obtained for the upregulated miRNAs in the cluster A1 (Figure S3E) and 708 target mRNAs were obtained for the upregulated miRNAs in the cluster B2 (Figure S3F). The common miRNAs between the differential expressed miRNAs and target miRNA and the common mRNAs between the differential expressed mRNAs and target mRNAs were selected. According to the above lncRNAs, miRNAs, and mRNAs, an interaction network was built that summarized potential molecular characteristics of different tumor immunophenotypes (Figure S3G).

### 3.8 Identification of the hub gene

**FIGURE 6:**
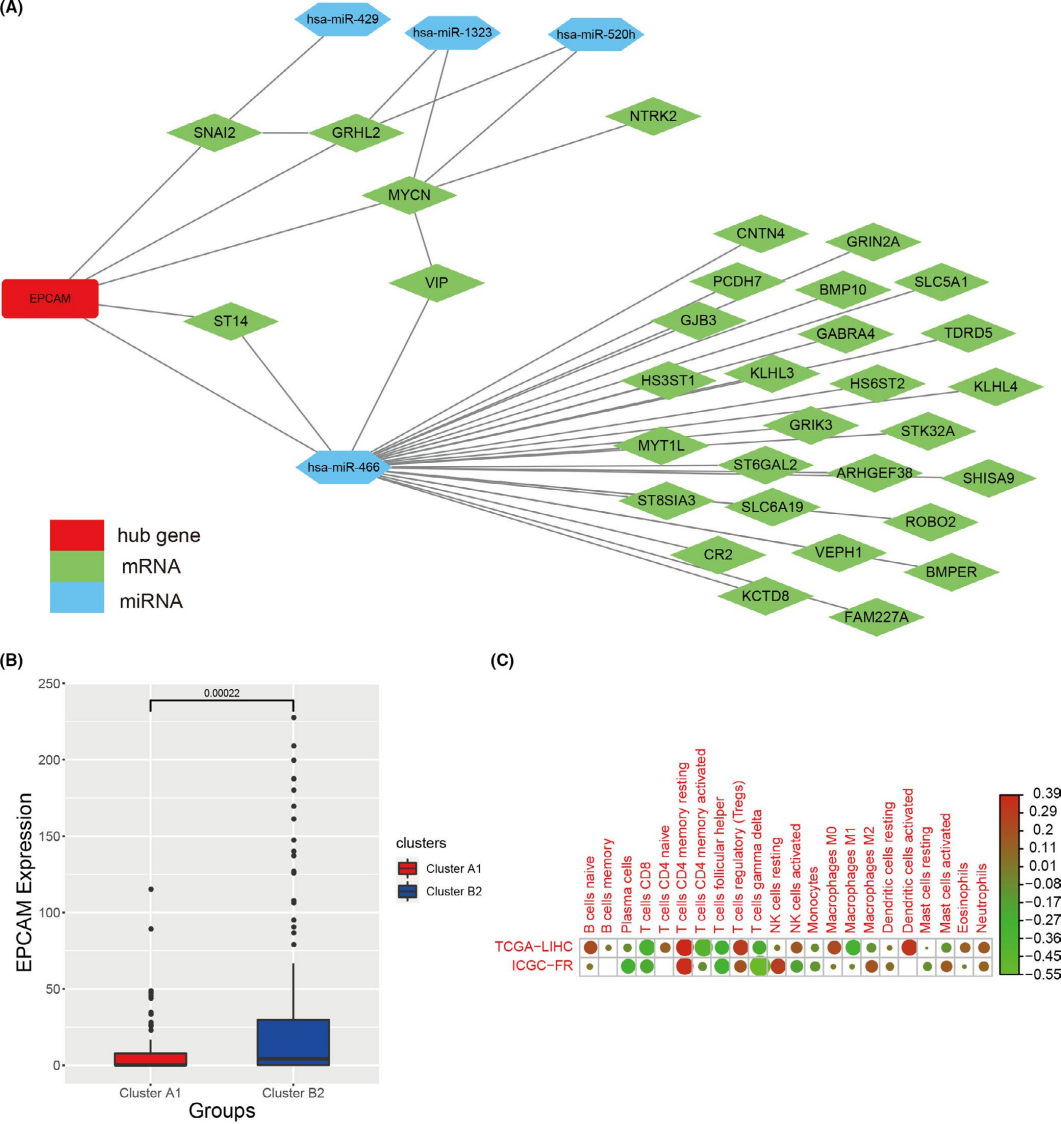
The analysis of the EPCAM gene. (A) The EPCAM related mRNAs and miRNAs. The red represented the hub gene, the green represented mRNAs, and the blue represented miRNAs. (B) The difference of EPCAM expression level between cluster A1 and cluster B2. (C) Correlation analysis between immune infiltrating cells and EPCAM expression level

In order to identify the key gene in the mRNAs–miRNAs–lncRNAs network, we used the cytohubba plug‐in of the Cytoscape and found that epithelial cell adhesion molecule (EPCAM) acted as a vital node within the network, which indicated that the EPCAM played an important influence on the difference of the immunophenotypes. The relevant network of EPCAM was visualized by the Cytoscape (Figure 6A). Moreover, EPCAM had a significantly higher expression in the cluster B2 than the cluster A1 (Figure 6B). To study the effect of other epigenetic factors on the EPCAM, the analysis of copy number variations and DNA methylation discovered that there was no significant difference of EPCAM between cluster A1 and cluster B2. The above results confirmed that the copy number variations and DNA methylation made no difference in the expression of the EPCAM. In addition, the correlation analysis showed that the EPCAM expression level had a negative association with T cells CD8, T cells CD4 memory activated, Macrophages M1, and T cells Folliculcar, which indicated this gene might provide a clue on the immunoregulation (Figure 6C).

### 3.9 Construction of the prognostic model

**FIGURE 7:**
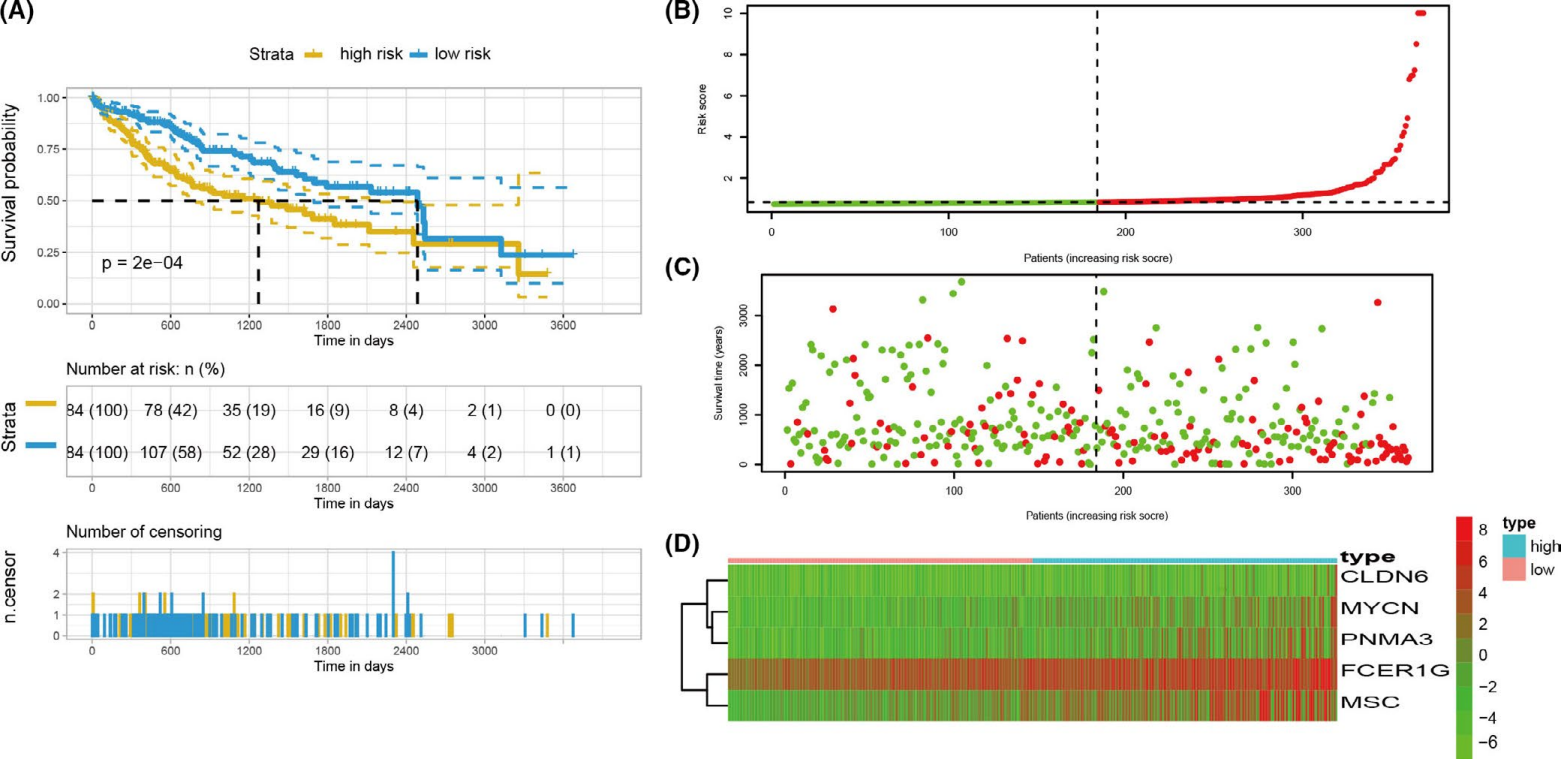
The construction of a prognostic model. (A) KM survival curve of patients with HCC in the training cohort. (B) Risk scores distribution of patients in the training cohort. The red points represented patients in the high‐risk group. The green points represented patients in the low‐risk group. (C) Survival time and survival status of patients in the training cohort. The red points represented patients with dead status. The green points represented patients with alive status. (D) The expression levels of prognostic genes in the training cohort. The red color represented that this gene was upregulated in patients and the green color represented that this gene was downregulated in patients. The depth of the color represented the level of expression. The “high” represented patients in the high‐risk group and the “low” represented patients in the low‐risk group. KM, Kaplan–Meier; HCC, hepatocellular carcinoma

To explore the prognosis‐related genes from the differentially expressed genes between cluster A1 and cluster B2, the LASSO Cox regression was used to select the most relevant prognosis genes including CLDN6, MYCN, PNMA3, FCER1G, and MSC to build the prognostic model. As expected, the high‐risk group had a poorer prognosis than the low‐risk group (Figure 7A). Figure 7B showed the risk score of patients in the high‐risk group and low‐risk group. Figure 7C showed the survival time and survival status of patients in the high‐risk group and low‐risk group. Furthermore, compared with the low‐risk group, the prognosis‐related gene expression levels of high‐risk group were higher (Figure 7D). To test whether the prognosis model had a similar prognostic function in other dataset, we applied the prognostic model to an independent validation set (GSE14520). Patients in the GSE14520 were also divided into high‐risk group and low‐risk group and survival rates were calculated. Patients in the low‐risk group had a better prognosis than the high‐risk group, which was consistent with the consequence obtained from the training set (Figure S4). The receiver operator characteristic (ROC) curve was used to assess the prognosis model. For 1‐year, 3‐year, and 5‐year survival times, the area under the curve (AUC) values of the model in the training set were 0.691, 0.668, and 0.672, respectively. The sensitivity was 0.436, 0.428, and 0.867, respectively. The specificity was 0.903, 0.848, and 0.449, respectively (Figure S5A). For 1‐year, 3‐year, and 5‐year survival times, the AUC values of the model in the validation set were 0.643, 0.585, and 0.58, respectively. The sensitivity was 0.667, 0.565, and 0.469, respectively. The specificity was 0.634, 0.654, and 0.745, respectively (Figure S5B).

## 4 DISCUSSION

In the past few years, the prognosis and genetic alterations of HCC patients have been deeply and extensively explored. TP53 and LRP1B genetic markers have been shown to play a vital part in prognostic and were broadly used in prognostic signatures.^44^ Furthermore, some studies indicated that the TP53 and LRP1B mutations could influence the immune microenvironment of cancer. However, the potential molecular mechanisms of TP53 and LRP1B and different immunophenotypes are still unknown. In this research, according to the expressions of the TP53 and LPR1B, we built the different immune phenotypes with higher (cluster A1) or lower immune cell levels (cluster B2). In addition, the integrated analysis of multi‐omics information was performed to emphasize the differences of the genome and epigenetic patterns between cluster A1 and cluster B2. EPCAM was identified as a pivotal factor in regulating tumor immune phenotypes. In addition, based on the expressions of relevant immune candidate genes, we set up a prognostic model and proved its prognosis value.

Although many studies indicated that the TP53 and LRP1B could affect the prognosis of HCC patients, the impacts of TP53 and LRP1B on the immune were poorly understood. In the previous research, Cooks et. al demonstrated that colon cancer cells with TP53 mutation could release exosomes to change tumor immune status by reprogramming macrophages.^45^ The mutation of LRP1B was correlated with ameliorative immunotherapy outcomes for melanoma and non‐small cell lung cancer (NSCLC) patients. Patients with the mutation of LRP1B often had the enrichment of genes, which were associated with antigen processing and presentation and cell cycle checkpoints.^46^ Therefore, more researches are needed to explore the underlying relationship between two genes and immune cells of HCC. We applied correlation analysis and lasso analysis to filter the immune cells, which were associated with TP53 and LRP1B. This double screening made the result more precise.^47^

Multi‐omics data analysis has significant advantages to reveal potential correlations among multiple biology factors and the functional mechanisms. According to the multi‐omics data analysis, we found that the EPCAM was the key gene to regulate the immune microenvironment. The expression level of EPCAM was considered to be closely associated with clinical outcomes in HCC. EPCAM has high expressions in numerous human cancers, which originate from epithelial.^48^ However, the function and the expression regulation of EPCAM are still unknown. It has been shown that recurrence of HCC was promoted, at least partly, by cancer stem cells (CSCs). CSCs contained specific biomarkers like EPCAM, which were participated in their effect to escape the immune system, to facilitate tumor growth and to generate colonies.^49^ The IFN‐γ derived from NK cells could promote HCC occurrence and development by the EPCAM‐EMT axis in the HBs‐Tg mice, indicating the significance of congenital immunity in the pathogenesis of HBV‐related HCC.^50^ Furthermore, based on the upregulated of CEACAM1 expression level, EPCAM liver CSCs could control NK cell‐mediated cytotoxicity.^51^

Finally, based on differentially expressed genes among cluster A1 and cluster B2, we established a prognostic model, including CLDN6, MYCN, PNMA3, FCER1G, and MSC. The CLDN6 gene acted as the oncogene in HCC and enhanced cancer cell invasion, migration, and proliferation through EGFR/AKT/mTOR signaling pathway.^52^ In neuroblastoma (NB) patients, the MYCN, as the potential biomarker, could predict the therapeutic efficacy susceptibility of NK cell‐mediated immunotherapy.^53^ In addition, the gene sets of immune‐associated pathways were usually enriched in renal cell carcinoma (RCC) patients, which had highly expressed FCER1G.^54^ These indicated that immune‐associated pathways could affect the prognosis of HCC patients.

We also recognized this study still had limitations. The somatic mutations, DNA methylation, and CNV variation failed to provide additional clues for the differential expression of the hub gene. In addition, there are no protein expression data of EPCAM, its different expression at the protein level cannot be analyzed. This result is needed to be further verified by the vast amounts of data.

## 5 CONCLUSION

In summary, we integrated multi‐omics information to demonstrate that TP53 and LRP1B served as the critical genes associated with the immune phenotypes and this process was meditated through the EPCAM. The immune‐related model was an important predictor to predict the prognosis of the HCC patients and may provide better insights into the immunological therapy.

## CONFLICT OF INTEREST

The authors have declared that no competing interest exists.

## AUTHOR CONTRIBUTIONS

Lili Wu and Yifen Shi designed the study and revised the manuscript. Jie Cao analyzed the data. Xin Lei interpreted the results of the experiments. Liang Shi contributed to the writing of the manuscript. All authors read and approved the final manuscript.

## ETHICAL APPROVAL STATEMENT

Not applicable.

## Supporting information


FIGURE S1

FIGURE S2

FIGURE S3

FIGURE S4

FIGURE S5

TABLE S1

TABLE S2

TABLE S3

## Data Availability

The data that support the findings of this study are available from the corresponding author or the first author upon reasonable request.
