# Supplementary figures and images for: Multi‐omics data identified TP53 and LRP1B as key regulatory gene related to immune phenotypes via EPCAM in HCC
Source: Cancer Med. 2022 Feb 12;11(10):2145–58. doi: 10.1002/cam4.4594 (PMC9119357; doi:10.1002/cam4.4594)

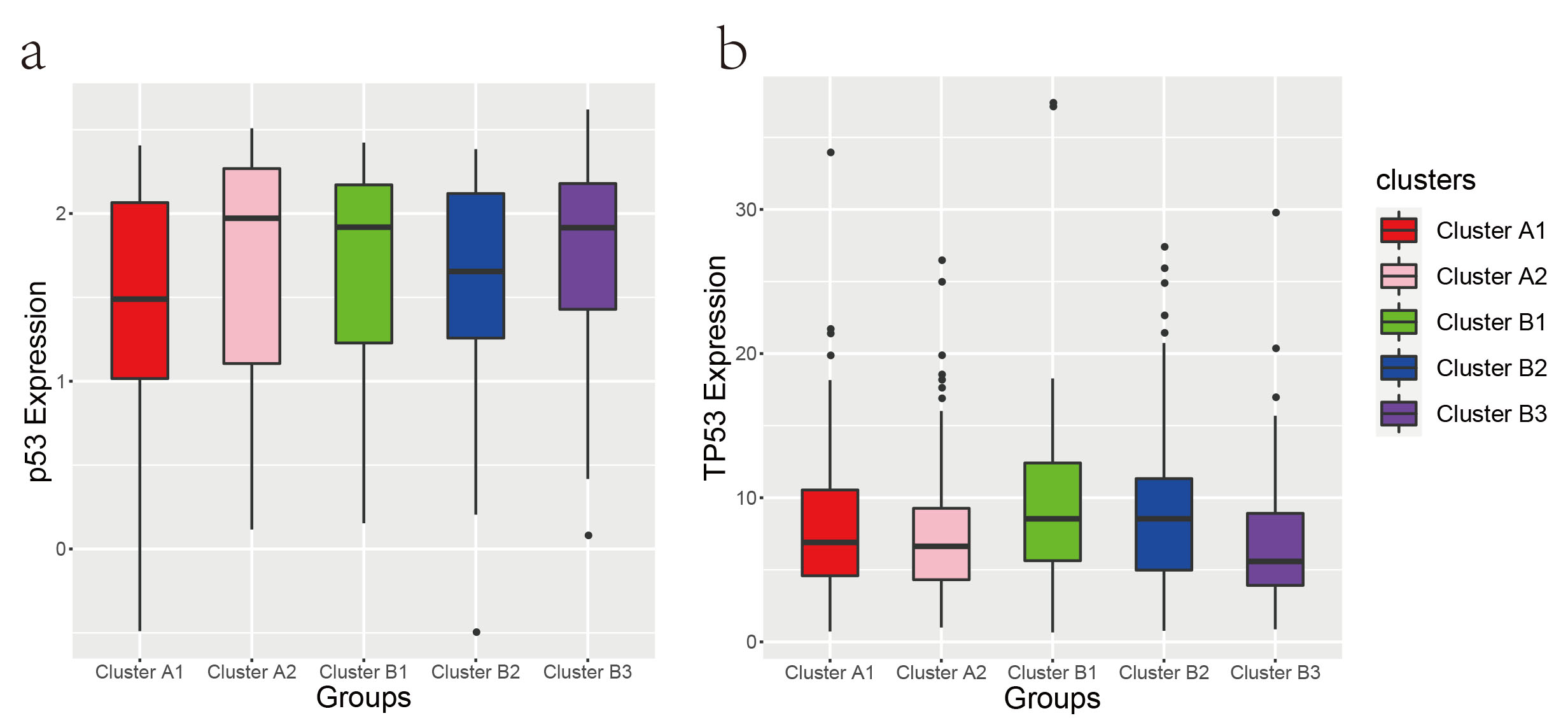

Supplement: Supplementary file 1 — FIGURE S1 [file CAM4-11-2145-s006.jpg]

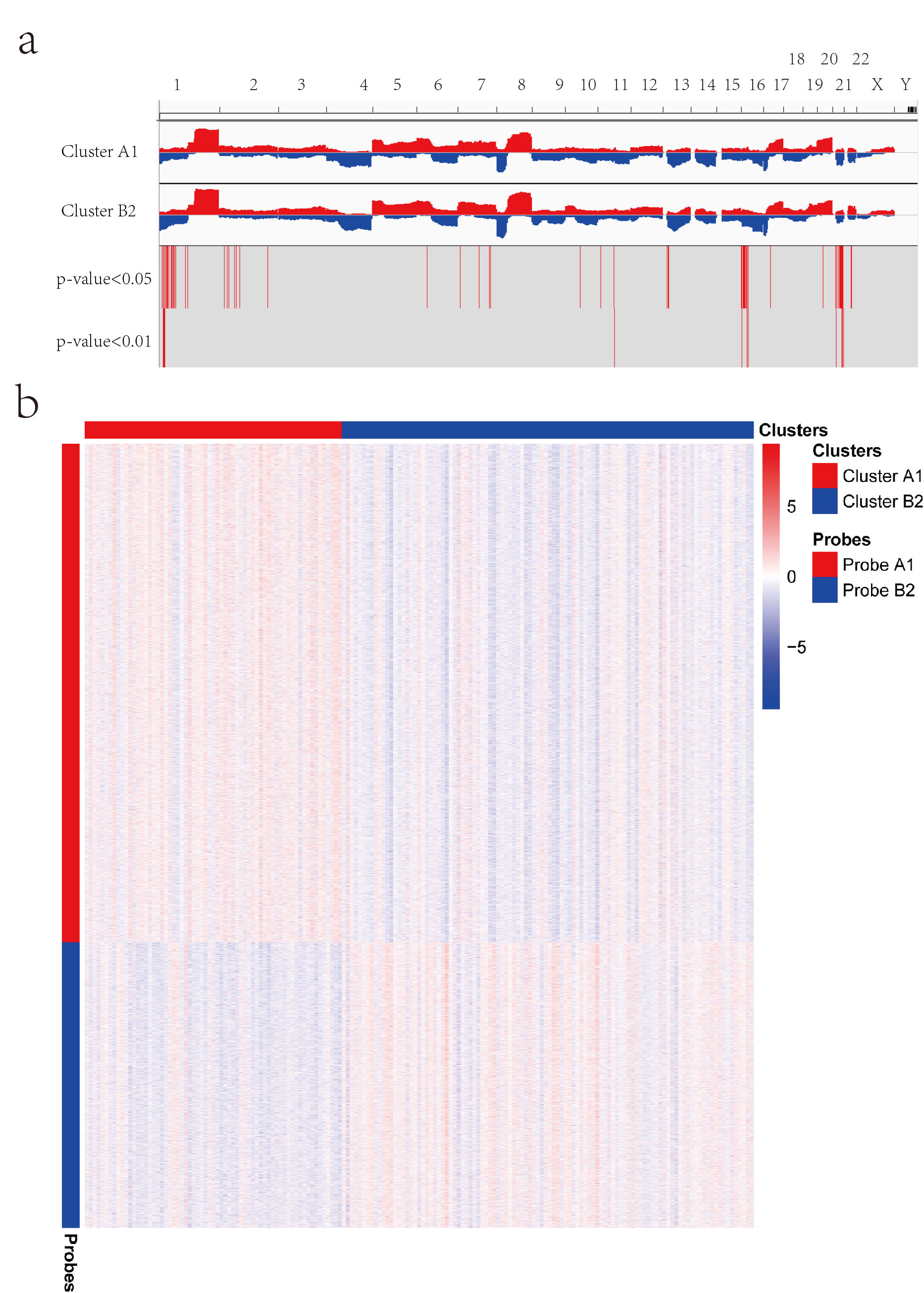

Supplement: Supplementary file 2 — FIGURE S2 [file CAM4-11-2145-s005.jpg]

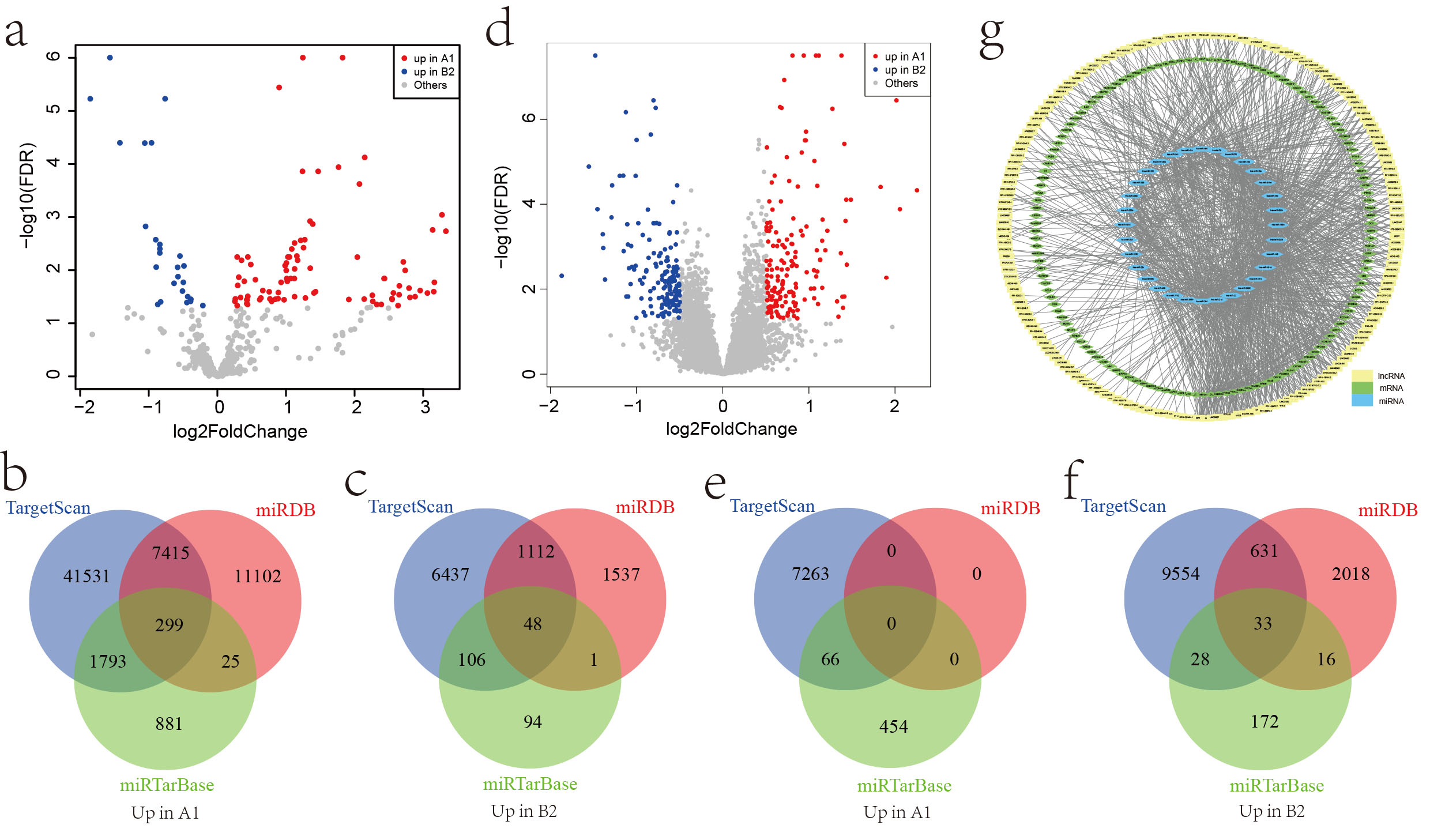

Supplement: Supplementary file 3 — FIGURE S3 [file CAM4-11-2145-s001.jpg]

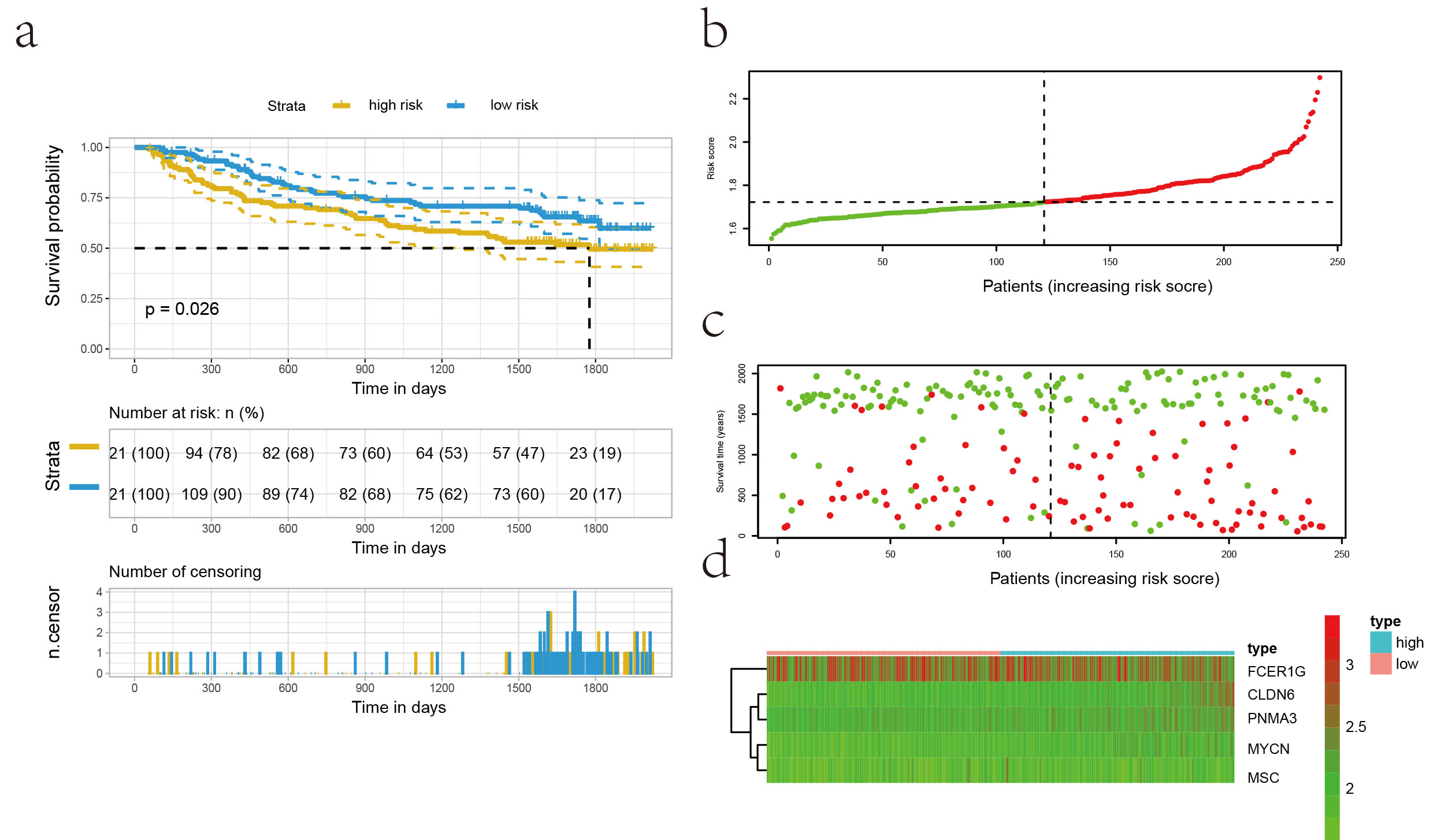

Supplement: Supplementary file 4 — FIGURE S4 [file CAM4-11-2145-s007.jpg]

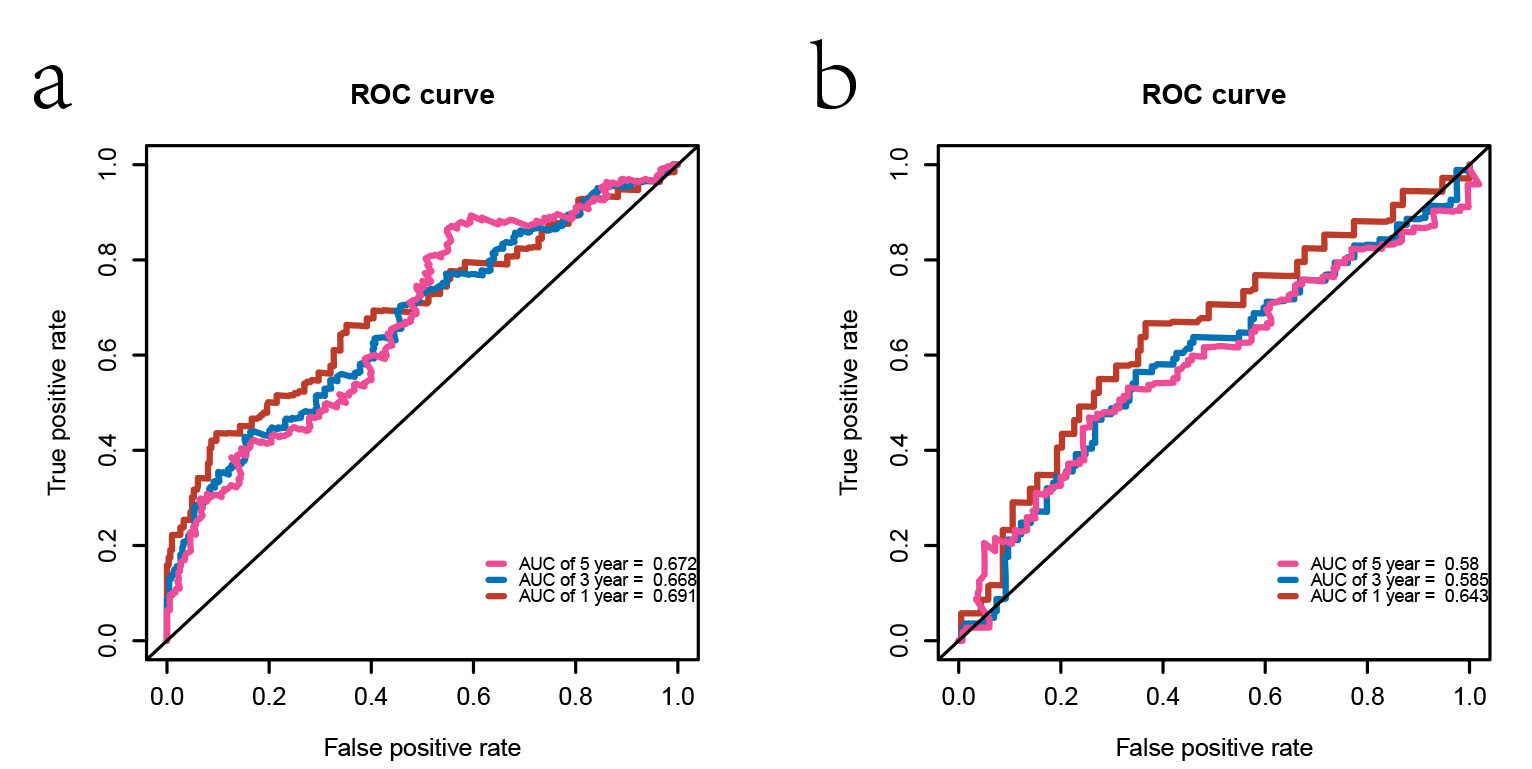

Supplement: Supplementary file 5 — FIGURE S5 [file CAM4-11-2145-s002.jpg]
