# Supplementary material for: Multi‐omics data identified TP53 and LRP1B as key regulatory gene related to immune phenotypes via EPCAM in HCC
Source: Cancer Med. 2022 Feb 12;11(10):2145–58. doi: 10.1002/cam4.4594 (PMC9119357; doi:10.1002/cam4.4594)
Supplement: Supplementary file 6 — TABLE S1 [file CAM4-11-2145-s003.docx]

| **Table S1. The tumor sample of the datasets** | |
| --- | --- |
|  | **Tumor** |
| TCGA-LIHC | 374 |
| ICGC-JP | 229 |
| ICGC-FR | 161 |
| GSE14520 | 247 |
