# Supplementary material for: Multi‐omics data identified TP53 and LRP1B as key regulatory gene related to immune phenotypes via EPCAM in HCC
Source: Cancer Med. 2022 Feb 12;11(10):2145–58. doi: 10.1002/cam4.4594 (PMC9119357; doi:10.1002/cam4.4594)
Supplement: Supplementary file 7 — TABLE S2 [file CAM4-11-2145-s004.docx]

| **Table S2. The stage and gender of HCC patients** | | | |
| --- | --- | --- | --- |
|  | **parameter** | **type** | **N (%)** |
| TCGA | Stage | T1 | 183(48.93%) |
|  |  | T2 | 95(25.4%) |
|  |  | T3 | 80(21.39%) |
|  |  | T4 | 13(3.48%) |
|  |  | NA | 3(0.81%) |
|  | Gender | Male | 253(67.65%) |
|  |  | Female | 121(32.35%) |
| ICGC-FR | Gender | Male | 127(78.88%) |
|  |  | Female | 34(21.12%) |
| ICGC-JP | Gender | Male | 168(73.36%) |
|  |  | Female | 61(26.64) |
| GSE14520 | Gender | Male | 211(85.42%) |
|  |  | Female | 31(12.55%) |
|  |  | NA | 5(2.02%) |
