# Supplementary material for: Multi‐omics data identified TP53 and LRP1B as key regulatory gene related to immune phenotypes via EPCAM in HCC
Source: Cancer Med. 2022 Feb 12;11(10):2145–58. doi: 10.1002/cam4.4594 (PMC9119357; doi:10.1002/cam4.4594)
Supplement: Supplementary file 8 — TABLE S3 [file CAM4-11-2145-s008.docx]

| **Table S3. The univariate analysis of the differentially expressed genes** | | |
| --- | --- | --- |
| **id** | **HR** | **pvalue** |
| ZNF488 | 21.90216 | 2.33E-08 |
| MYCN | 1.091803 | 6.56E-07 |
| IGLON5 | 1.283801 | 2.20E-06 |
| MYH6 | 5.676175 | 4.70E-06 |
| SBK3 | 1.451964 | 7.31E-06 |
| MSC | 1.01011 | 2.13E-05 |
| SERPINB5 | 2.140818 | 3.09E-05 |
| TRIM16L | 1.026989 | 5.43E-05 |
| ANKRD33 | 1.145377 | 7.13E-05 |
| CLDN6 | 1.140232 | 9.40E-05 |
| KRT17 | 1.023376 | 0.000161 |
| DNAJC5G | 3.048418 | 0.000203 |
| FCER1G | 1.004587 | 0.000215 |
| MMP3 | 1.173381 | 0.000358 |
| CD7 | 1.012945 | 0.000453 |
| CRISP2 | 1.460722 | 0.000463 |
| WDR87 | 1170.382 | 0.000532 |
| FGF11 | 226.0844 | 0.000799 |
| FLNC | 1.027194 | 0.000839 |
| PNMA3 | 1.047135 | 0.001045 |
| CACNA1B | 3.063782 | 0.001094 |
| KISS1R | 1.23744 | 0.001247 |
| P2RY4 | 1.342188 | 0.001483 |
| NR0B1 | 1.077033 | 0.002262 |
| DCAF12L2 | 7.380341 | 0.002364 |
| FBXW10 | 1.228345 | 0.003039 |
| PYDC1 | 1.145987 | 0.003289 |
| FMN2 | 1.601316 | 0.003414 |
| FCGR3A | 1.009797 | 0.003595 |
| KLHL4 | 1.599988 | 0.003752 |
| FCGR1A | 1.107005 | 0.004187 |
| SLC22A12 | 1.015555 | 0.004187 |
| FCGR1B | 1.960125 | 0.004416 |
| RP11-766F14.2 | 25.85282 | 0.004673 |
| KCNH2 | 1.036861 | 0.004711 |
